# Allogeneic Anti-BCMA CAR T Cells Are Superior to Multiple Myeloma-derived CAR T Cells in Preclinical Studies and May Be Combined with Gamma Secretase Inhibitors

**DOI:** 10.1158/2767-9764.CRC-21-0157

**Published:** 2022-03-23

**Authors:** Ana M. Metelo, Agnieszka Jozwik, Le Anh Luong, Delaney Dominey-Foy, Charlotte Graham, Charlotte Attwood, Shafqat Inam, Alan Dunlop, Katy Sanchez, Kirsty Cuthill, Carmel Rice, Matthew Streetly, Trevor Bentley, Bijan Boldajipour, Cesar Sommer, Barbra Sasu, Reuben Benjamin

**Affiliations:** 1 Comprehensive Cancer Center, Faculty of Life Sciences and Medicine, King's College London, London, United Kingdom.; 2 King's College Hospital NHS Foundation Trust, London, United Kingdom.; 3 Guy's and St. Thomas’ NHS Foundation Trust, London, United Kingdom.; 4 Allogene Therapeutics Inc., South San Francisco, California.; 5 Pfizer Inc., South San Francisco, California.

## Abstract

Multiple myeloma remains an incurable plasma cell malignancy despite the rapidly evolving treatment landscape. Chimeric antigen receptor T cells targeted against BCMA have recently shown great promise in relapsed refractory multiple myeloma; however, all patients ultimately still progress from their disease. Lack of CAR T-cell persistence, impaired T-cell fitness in autologous CAR T-cell products and the presence of an immunosuppressive bone marrow (BM) microenvironment are contributory factors to treatment failure. We generated anti-BCMA CAR T cells from healthy donors (HD) and patients with multiple myeloma at different stages of disease to compare their T-cell profile, fitness, and cytotoxic activity in preclinical studies. We also used an *ex vivo* assay with multiple myeloma BM biopsies from distinct genomic subgroups to test the efficacy of HD-derived CAR T cells in a clinically relevant model. HD volunteers showed increased T-cell counts, higher CD4/CD8 ratio, and expanded naïve T-cell population compared with patients with multiple myeloma. After anti-BCMA CAR T-cell production, patients with relapsed multiple myeloma had lower frequencies of CAR^+^ T cells, decreased central memory phenotype, and increased checkpoint inhibitory markers compared with HD-derived products, which compromised their expansion and cytotoxicity against multiple myeloma cells *in vitro*. Importantly, HD-derived CAR T cells efficiently killed primary multiple myeloma cells within the BM microenvironment of different multiple myeloma genomic subgroups and their cytotoxic activity could be boosted with gamma secretase inhibitors. In conclusion, allogeneic anti-BCMA CAR T cells are a potential therapeutic strategy for patients with relapsed multiple myeloma and should be further developed in the clinic.

**Significance:**

Multiple myeloma is an incurable cancer of the plasma cells. A new therapy with anti-BCMA CAR T cells — the patient's own T cells genetically engineered to find and kill myeloma cancer cells — has shown encouraging results. Unfortunately, patients still relapse. In this study, we propose to use T cells from HD volunteers, which have a stronger T-cell fitness, higher cancer killing capacity, and are ready to be administered when needed.

## Introduction

Multiple myeloma is a plasma cell malignancy with a heterogeneous genomic profile that remains incurable despite the use of therapies such as alkylating agents, proteasome inhibitors, immunomodulatory drugs, and anti-CD38–targeted mAbs (1, 2). Whilst the overall survival of patients with standard-risk multiple myeloma can approach 10 years, those with high-risk cytogenetic abnormalities such as t(4;14), del(17/17p), t(14;16), t(14;20), and gain(1q) have a significantly worse prognosis of 2–3 years (3). Consequently, while the therapeutic landscape of multiple myeloma has increased significantly in the last ten years, there is still an urgent need for novel and effective therapies.

Chimeric antigen receptor (CAR) T cells have recently emerged as a very promising therapeutic strategy in cancer with anti-CD19 CAR T cells now approved for the treatment of relapsed diffuse large B-cell lymphoma, mantle cell lymphoma, and pediatric/young adult B-cell acute lymphoblastic leukemia (4–13). The main target for CAR T-cell therapy in multiple myeloma has been B-cell maturation antigen (BCMA), based on its high specificity and broad expression across multiple myeloma cells irrespective of genomic subtype (14, 15). BCMA is only expressed on late B cells, normal plasma cells, and multiple myeloma cells, and not on hematopoietic stem cells, which makes it an ideal cancer associated antigen to target by immunotherapy (14, 16–18). Different anti-BCMA CAR T-cell products have been developed over recent years and several clinical trials have shown impressive response rates to anti-BCMA CAR T-cell therapy in patients with relapsed refractory multiple myeloma (19–21). However the median duration of response observed in these trials ranged from 8.8 to 22 months following CAR T infusion.

While the mechanisms of CAR T-cell resistance in multiple myeloma are still under investigation (22, 23), downregulation of BCMA expression and lack of CAR T-cell persistence, due to CAR T-cell differentiation and exhaustion, are potential contributory factors. Clinical trials of autologous anti-BCMA CAR T cells in multiple myeloma have shown differences in T-cell profiles between responders and nonresponders, with T-cell exhaustion and terminal differentiation compromising the efficacy of the CAR T-cell products (24–26). Therefore an alternative strategy is to use allogeneic healthy donor (HD)-derived CAR T cells since they are generated from young HDs, whose T cells are likely to have a more favorable phenotype, superior immune fitness and cytotoxic ability (27).

In this study, we compared the T-cell profile and activity of anti-BCMA CAR T cells derived from HDs with those generated from patients with multiple myeloma of different genomic subtypes and disease stages, including patients who were eligible for CAR T-cell clinical trials. The cytotoxicity of anti-BCMA CAR T cells was evaluated in primary bone marrow (BM) cultures from patients with multiple myeloma enabling the potential immunosuppressive effects of the BM microenvironment to be explored. In addition, the effects of enhancing BCMA expression, using a gamma secretase inhibitor (GSI), on CAR T-cell cytotoxicity were investigated.

## Materials and Methods

### Patients and HD Volunteers

Peripheral blood and BM samples from HD volunteers and patients with multiple myeloma were obtained from the Haemato-Oncology Tissue Bank at King's College London under the terms of the research ethics protocol reference HR-17/18–5515 in accordance with the Declaration of Helsinki and after approval by an institutional review board and with the Human Tissue Authority license number 12223. Written informed consent forms were signed by all the patients prior to sample collection.

### Anti-BCMA CAR T-Cell Production

Peripheral blood mononuclear cells (PBMC) were isolated using Histopaque 1077 density gradient media (Sigma, catalog no. RNBG8589) and centrifuged at 2,000 rpm for 30 minutes without any brake. PBMCs present in the cellular buffy coat were collected, washed twice with sterile PBS, and frozen down for future processing and analysis.

On day 0 of production, PBMC samples were thawed and T cells isolated using the EasySep Human T Cell Enrichment Kit (StemCell Technologies, 18M98363). Enriched T cells were plated in complete X-Vivo 15 media (with 5% FBS, 1% nonessential amino acids, 1% sodium pyruvate, and 20 mmol/L HEPES), supplemented with recombinant human IL2 IS premium grade (20 ng/μL; Miltenyi Biotec, catalog no. 130–097–745) and activated with human magnetic CD3/CD28 beads (Thermo Fisher Scientific, catalog no. 11131D). After 48 hours, activated T cells were transduced with a lentiviral construct encoding the BCMA-5 R2 CAR (28) containing an anti-BCMA scFv with 4–1BB and CD3z domains, and the media further supplemented with recombinant human IL2. On day 5, human CD3/CD28 beads were removed from each sample using an EasySep magnet and the individual samples were plated in complete X-Vivo 15 media with human IL2. Cells were cultured for 9 days and frozen on day 14. Untransduced T cells (UT) from each of the donors were used as negative controls and underwent the same manufacturing steps as the corresponding anti-BCMA CAR T cells with the exception of the lentiviral transduction step.

### T-Cell Immunostaining

T cells were stained with the following fluorochrome-conjugated mouse monoclonal anti-human antibodies for 20 minutes at room temperature: anti-CD25 FITC (BioLegend, catalog no. 302604, RRID:AB_314274), anti-CD38 FITC (BioLegend, catalog no. 303504, RRID:AB_314356), anti-TIM3 PerCP-Cy5.5 (BioLegend, catalog no. 345015), anti-CD4 Pacific Blue (BioLegend, catalog no. 317429), anti-CD45RO Brilliant Violet 605 (BioLegend, catalog no. 304238), anti-TIGIT Brilliant Violet 605 (BioLegend, catalog no. 372711), anti-PD1 Brilliant Violet 650 (BioLegend, catalog no. 329949), anti-CD3 Brilliant Violet 711 (BioLegend, catalog no. 317328), anti-CD101 PE (BioLegend, catalog no. 331011, RRID:AB_2716106), anti-LAG3 PE Dazzle (BioLegend, catalog no. 369331), anti-CD62 L PE Dazzle (BioLegend, catalog no. 304842), anti-CD8 PE-Cy7 (BioLegend, catalog no. 301012), and the anti-BCMA CAR idiotype APC (Allogene Therapeutics). T cells were then washed with PBS and stained with the e780 fixable viability dye (Thermo Fisher, catalog no. 65–0865–14) for 30 minutes at 4°C. All the samples were fixed using BD stabilizing fixative (BD Biosciences, catalog no. 339860) for further FACS analysis.

### BM Sample Processing

Fresh BM samples were transferred into 50 mL Falcon tubes and incubated with Pharm lysis buffer (BD Biosciences, catalog no. 555899) for 15 minutes at room temperature. The samples were then centrifuged and washed twice with sterile PBS, before passing through a 100-μm mesh (Miltenyi Biotec). The samples were then incubated in RPMI1640 complete media (with 10% FBS, 1× penicillin/streptomycin, and 1× l-glutamine) and stored in the tissue culture incubator at 37°C, 5% CO_2_. Overall cell viability and multiple myeloma cell percentage were then measured by FACS as described below. BM samples were always used fresh and the available volume varied from sample to sample, which limited the number of samples used in specific assays.

**FIGURE 1:**
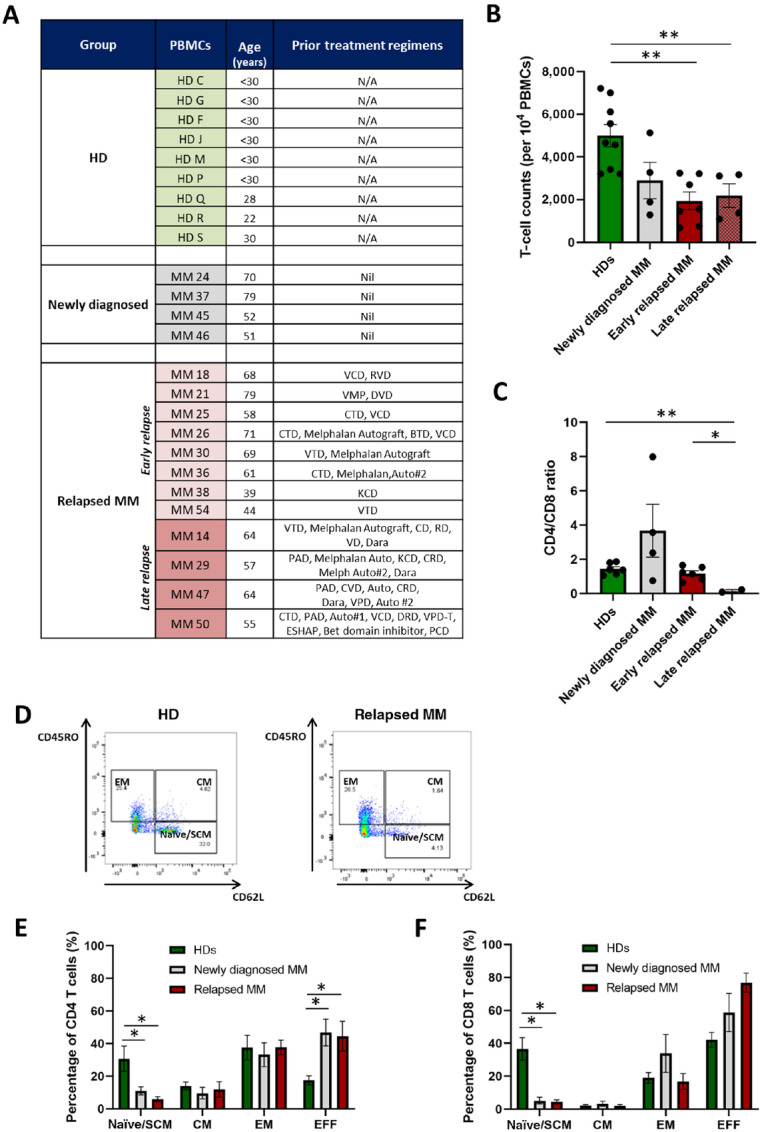
HD volunteers have higher T-cell counts, increased CD4/CD8 ratio, and an expanded naïve T-cell population compared with patients with multiple myeloma. **A,** Clinical features of HD volunteers and patients with multiple myeloma (MM) used in the study to generate PBMCs, including age and prior treatment regimens. Three main groups were studied: (i) HD volunteers with no underlying disorders and age < 30 (HD), newly diagnosed multiple myeloma patients not yet exposed to any treatment regimen, and relapsed multiple myeloma patients, who have undergone several lines of treatment as described. The third group is subdivided into early relapse (≤3 prior lines of therapy) and late relapse (>3 prior lines of therapy). **B,** T-cell counts per 10,000 live PBMCs after PBMC isolation from peripheral blood: HD (*n* = 9), newly diagnosed MM (*n* = 4), early relapsed MM (*n* = 9), and late relapsed MM (*n* = 4). **C,** CD4/CD8 T-cell ratios from the four groups analyzed above. **D,** Representative FACS plots describing the T-cell memory phenotype from a HD subject versus a relapsed multiple myeloma patient. Cells were previously gated on live CD3^+^ T cells and based on the expression of CD45RO and CD62L were characterized into: naïve/stem cell memory (SCM), central memory (CM), and effector memory (EM) T cells. CD45RO^−^ and CD62L^−^ cells were quantified as effector T cells. Memory phenotype of CD4 (**E**) and CD8 T cells (**F**) present in HD (*n* = 6), newly diagnosed multiple myeloma (*n* = 4), and relapsed multiple myeloma patients (*n* = 8, including early and late rMM patients), quantified by FACS based on CD45RO and CD62L expression. Data represent mean values ± SEM. *P < 0.05; **P < 0.005. Statistical analysis was performed using two-tailed unpaired *t* test. Abbreviations: Auto, autologous stem cell transplant/autograft; BTD, Bendamustine, Thalidomide, Dexamethasone; CD, Cyclophosphamide, Dexamethasone; CRD, Cyclophosphamide, Lenalidomide, Dexamethasone; CTD, Cyclophosphamide, Thalidomide, Dexamethasone; CVD or VCD, Cyclophosphamide, Velcade, Dexamethasone; Dara, Daratumumab; DVD, Daratumumab, Velcade, Dexamethasone; ESHAP, Etoposide, Solu-medrone, High-dose cytarabine, Cisplatin; KCD, Carfilzomib, Cyclophosphamide, Dexamethasone; Nil, no prior treatment regimen; PAD, Velcade, Adriamycin, Dexamethasone; RD – Lenalidomide, Dexamethasone; RVD, Lenalidomide, Velcade, Dexamethasone; VD, Velcade, Dexamethasone; VMP, Velcade, Melphalan, Prednisolone; VPD, Velcade, Panobinostat, Dexamethasone; VTD, Velcade, Thalidomide, Dexamethasone.

### *Ex Vivo* BM Cytotoxicity Assay

BM samples were stained with a multiple myeloma antibody panel to quantify BM cell viability and the percentage of multiple myeloma primary cells using the following mouse monoclonal anti-human antibodies: anti-CD56 BV605 (BioLegend, catalog no. 318334, RRID:AB_2561912), anti-BCMA PE (BioLegend, catalog no. 357504), anti-CD19 PE-Cy7 (BioLegend, catalog no. 302216), anti-CD138 APC (BioLegend, catalog no. 356506, RRID:AB_2561880), and anti-CD38 APC-Cy7 (BioLegend, catalog no. 356616), and anti-CD45 FITC (BD Biosciences, catalog no. 345808, RRID:AB_2732010). The samples were incubated with e450 Viability Dye (Thermo Fisher, catalog no. 65–0863–14) and fixed using the BD stabilizing fixative for further FACS analysis.

On the basis of the number of multiple myeloma primary cells present in each BM sample, HD-derived anti-BCMA CAR T cells or UT cells were cocultured with BM at effector:target (E:T) ratios of 10:1, 5:1, 2.5:1, and 1:1, in RPMI1640 complete media. BM on its own was used to quantify the spontaneous multiple myeloma cell lysis. The human acute lymphocytic leukemia cell line REH and the BCMA-expressing isogenic cell line (REH-BCMA) were used as negative and positive controls respectively. After 4 hours of coculture, samples were stained with the multiple myeloma antibody panel as described above. For the combination studies, the GSI PF-03084014 hydrobromide (Sigma Aldrich, catalog no. PZ0298–5MG) was used. The BM samples were pretreated with the GSI drug for 1 hour at 37°C before anti-BCMA CAR T cells or UT cells were added to the cocultures.

### Cytotoxicity Assay with U266 Cell Line

Anti-BCMA CAR T cells and UT cells produced from HD volunteers and patients with late relapsed multiple myeloma were thawed and incubated overnight in complete RPMI1640 media supplemented with human IL2 IS premium grade (20 ng/μL; Miltenyi Biotec, catalog no. 130–097–745). The human multiple myeloma cell line U266 was kindly supplied by Dr. Kwee Yong from University College of London (London, United Kingdom) in May 2017. FISH has been conducted to confirm U266 myeloma karyotype. No further cell line authentication has been performed. The cell line was thawed and maintained in culture for a maximum period of 24 passages and tested regularly for *Mycoplasma* using the EZ-PCR Mycoplasma Test Kit (Biological Industries, catalog no. 20–700–20). On days 0 and 4, the T cells were challenged with U266 multiple myeloma cells at an E:T ratio of 1:10. On day 8, cocultures were collected and stained with the multiple myeloma antibody panel before further analysis of U266 cell viability and T-cell expansion by FACS.

### FACS Acquisition and Data Analysis

Samples were run on a BD Flow Cytometer (BD LSRFortessa Fortessa Flow Cytometer, RRID:SCR_019601) using the BD FACSDiva software (BD FACSDiva Software, RRID:SCR_001456) for data acquisition. FlowJo v10.7 software (FlowJo, RRID:SCR_008520) was used for FACS data analysis and GraphPad Prism 8 (GraphPad Prism, RRID:SCR_002798) was used for data presentation and statistical analysis.

Multiple myeloma primary cells present in patient BM samples were identified using the following gating strategy: side scatter/CD45, CD138^pos^/CD38^high^, CD38^high^/CD45^low^ and CD56/CD19^neg^. The anti-BCMA CAR T-cell killing percentage was quantified as: [(% multiple myeloma cell lysis cocultured with anti-BCMA CAR T cells − % multiple myeloma cell lysis cocultured with UT cells)/% spontaneous multiple myeloma cell lysis].

### Data Availability Statement

The data generated in this study are available upon request from the corresponding author.

## Results

### HDs have Higher T-Cell Counts, CD4/CD8 T-Cell Ratio, and Naïve T-Cell Phenotype Compared with Patients with Multiple Myeloma

PBMCs were collected from 9 HDs and 16 patients with multiple myeloma, their immune profile assessed by flow cytometry and the antitumor efficacy of anti-BCMA CAR T-cell products generated from them compared. The median age of HDs was 26.7 years in contrast with 61.3 years for patients with multiple myeloma (Fig. 1A). Patients with multiple myeloma were categorized into newly diagnosed multiple myeloma (untreated, n = 4), early relapsed multiple myeloma (received ≤3 prior lines of therapy, *n* = 8), and late relapsed multiple myeloma (received >3 prior lines of therapy, *n* = 4). Details of treatment regimens received by patients with relapsed multiple myeloma are shown in Fig. 1A.

Following PBMC isolation, HD volunteers had the highest T-cell counts with a mean of 4,993 ± 523 T cells per 10^4^ PBMCs compared with 2,898 ± 853, 1,945 ± 416 and 2,190 ± 552 T cells per 10^4^ PBMCs (*P* < 0.005) for patients with newly diagnosed multiple myeloma, early relapsed multiple myeloma, and late relapsed multiple myeloma, respectively, as shown in Fig. 1B. CD4/CD8 ratio was variable in patients with newly diagnosed multiple myeloma (3.67 ± 1.27) but not significantly different from HDs (1.43 ± 0.1) or patients with early relapsed multiple myeloma (1.18 ± 0.2). However, CD4/CD8 ratio was significantly lower in the late relapsed multiple myeloma group compared with HDs (0.15 vs. 1.43 ± 0.1, *P* < 0.005; Fig. 1C).

T-cell memory phenotype, a surrogate marker of T-cell fitness (29–32), was characterized by FACS using CD45RO and CD62L cell surface markers as shown in Fig. 1D as a representative patient for each group. Analysis of CD4 T cells showed a significantly higher proportion of naïve/stem cell memory (SCM) phenotype in HDs (30.8 ± 7.7%) compared with newly diagnosed multiple myeloma (11.1 ± 2.4%, *P* < 0.05) or patients with relapsed multiple myeloma (6.0 ± 1.5%, *P* < 0.05; Fig. 1E). In addition, there were significantly fewer effector T cells (CD45RO^−^CD62L^−^) in HDs (17.5 ± 2.8%) compared with newly diagnosed multiple myeloma (46.8 ± 8.2%, *P* < 0.05) or patients with relapsed multiple myeloma (44.6 ± 9.1%, *P* < 0.05). No significant differences were seen in the percentages of central memory (CM) or effector memory (EM) T cells between the different groups. Analysis of the CD8 T-cell population (Fig. 1F) also showed a significantly higher proportion of naïve/SCM T cells in HDs (36.6 ± 6.8%) compared with newly diagnosed or patients with relapsed multiple myeloma (5.1 ± 2.0% and 4.4 ± 1.2%, respectively, *P* < 0.05) with no significant differences for the other T-cell populations.

### Anti-BCMA CAR T Cells Generated from HDs have a Higher Transduction Rate and Increased Proportion of Central Memory Cells Compared with CAR T Cells Derived from Patients with Relapsed Multiple Myeloma

**FIGURE 2:**
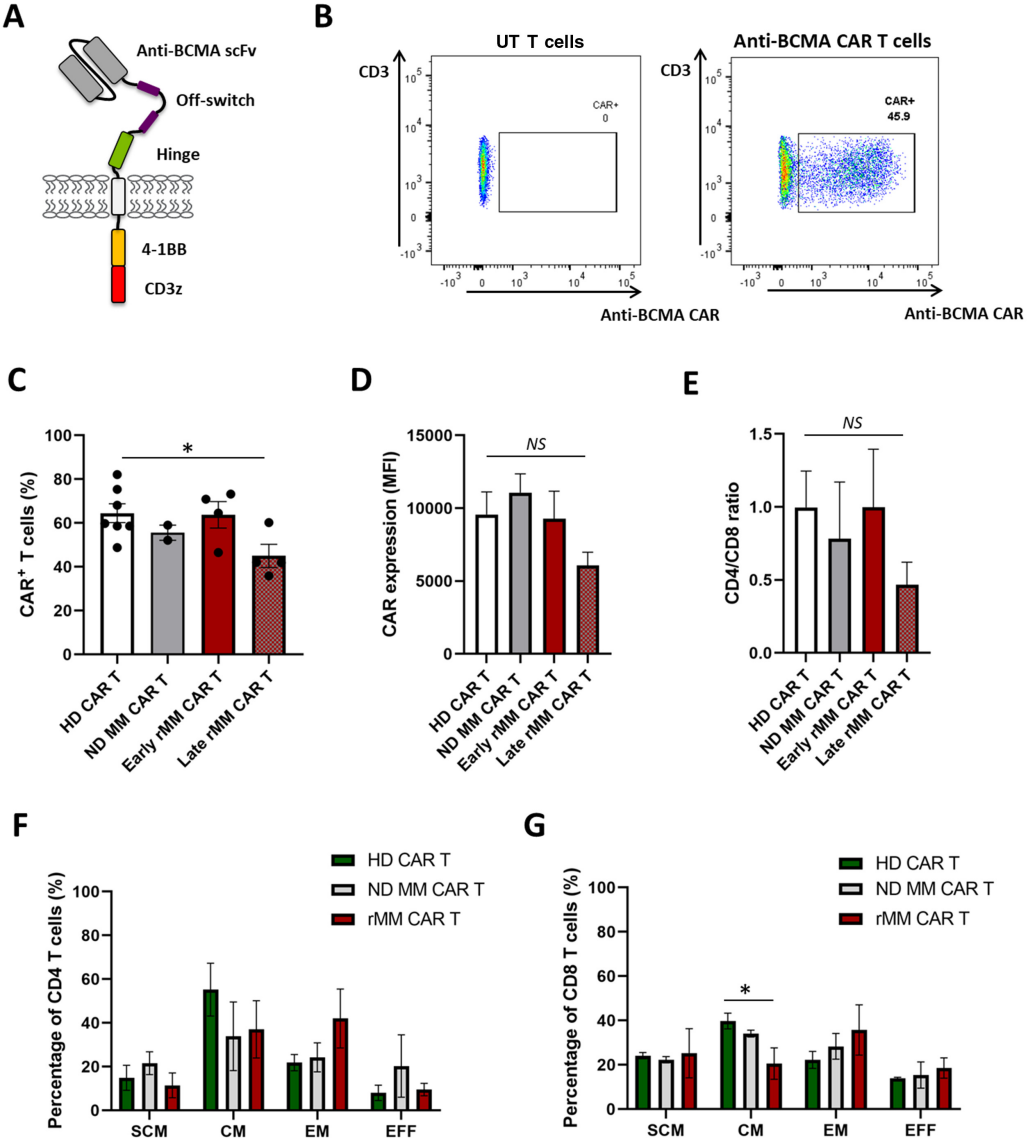
HD-derived anti-BCMA CAR T cells have a higher proportion of central memory CD8 T cells compared with multiple myeloma–derived anti-BCMA CAR T cells. **A,** Schematic diagram of the anti-BCMA CAR construct used, which includes an anti-BCMA scFv, a rituximab-based off-switch, CD8-based hinge and transmembrane region, 4–1BB co-stimulatory domain and CD3z intracellular signaling domain. **B,** Representative dot plots depicting anti-BCMA CAR expression on live CD3^+^ T cells by FACS after 14 days of anti-BCMA CAR T-cell production. UT cells were generated from the same donor. **C,** Percentage of anti-BCMA CAR^+^ T cells at the end of production generated from HD (*n* = 7), ND MM (*n* = 2), early rMM (*n* = 4), and late rMM patients (*n* = 4). **D,** Expression of anti-BCMA CAR (MFI) on CAR T cells generated as above. **E,** CD4/CD8 ratio of anti-BCMA CAR T cells generated as above. Memory phenotype of CD4 (**F**) and CD8 (**G**) anti-BCMA CAR T cells produced from HD (*n* = 3), newly diagnosed multiple myeloma (ND MM) (*n* = 2), and relapsed multiple myeloma (rMM) patients (*n* = 4, including early and late rMM patients). Data represent mean values ± SEM. *P < 0.05; NS, not statistically significant. Statistical analysis was performed using two-tailed unpaired *t* test.

To evaluate differences in phenotype and efficacy between HD-derived and multiple myeloma–derived CAR T cells, a second-generation fully human anti-BCMA CAR construct (ref. 28; Fig. 2A) was used to generate anti-BCMA CAR T cells from HDs and patients with multiple myeloma, subcategorized into newly diagnosed (ND), early relapsed and late relapsed multiple myeloma patients. FACS analysis of anti-BCMA CAR T cells after 14 days of manufacturing showed a higher percentage of CAR-transduced cells in HD-derived samples compared with late relapsed multiple myeloma samples (64.5 ± 4% vs. 45 ± 5.2%, *P* < 0.05), although CAR expression as measured by mean fluorescence intensity (MFI) was similar between the different groups (Fig. 2B–D). The CD4/CD8 ratio of the final CAR T-cell product was not significantly different between any of the groups (Fig. 2E). HD CAR T cells showed a higher percentage of central memory CD8 T cells compared with rMM CAR T cells (39.7 ± 4% vs. 20.5 ± 7%, *P* < 0.05) which may have an impact on their cytotoxic capacity. The proportions of stem cell memory, effector memory, and effector cells did not appear to be significantly different between HD, newly diagnosed multiple myeloma and relapsed multiple myeloma CAR T-cell products (Fig. 2F and G).

### HD-Derived anti-BCMA CAR T Cells Have a Less Dysfunctional Phenotype and Show Superior Cytotoxicity Compared with Late Relapsed Multiple Myeloma–Derived CAR T Cells *In Vitro*

**FIGURE 3:**
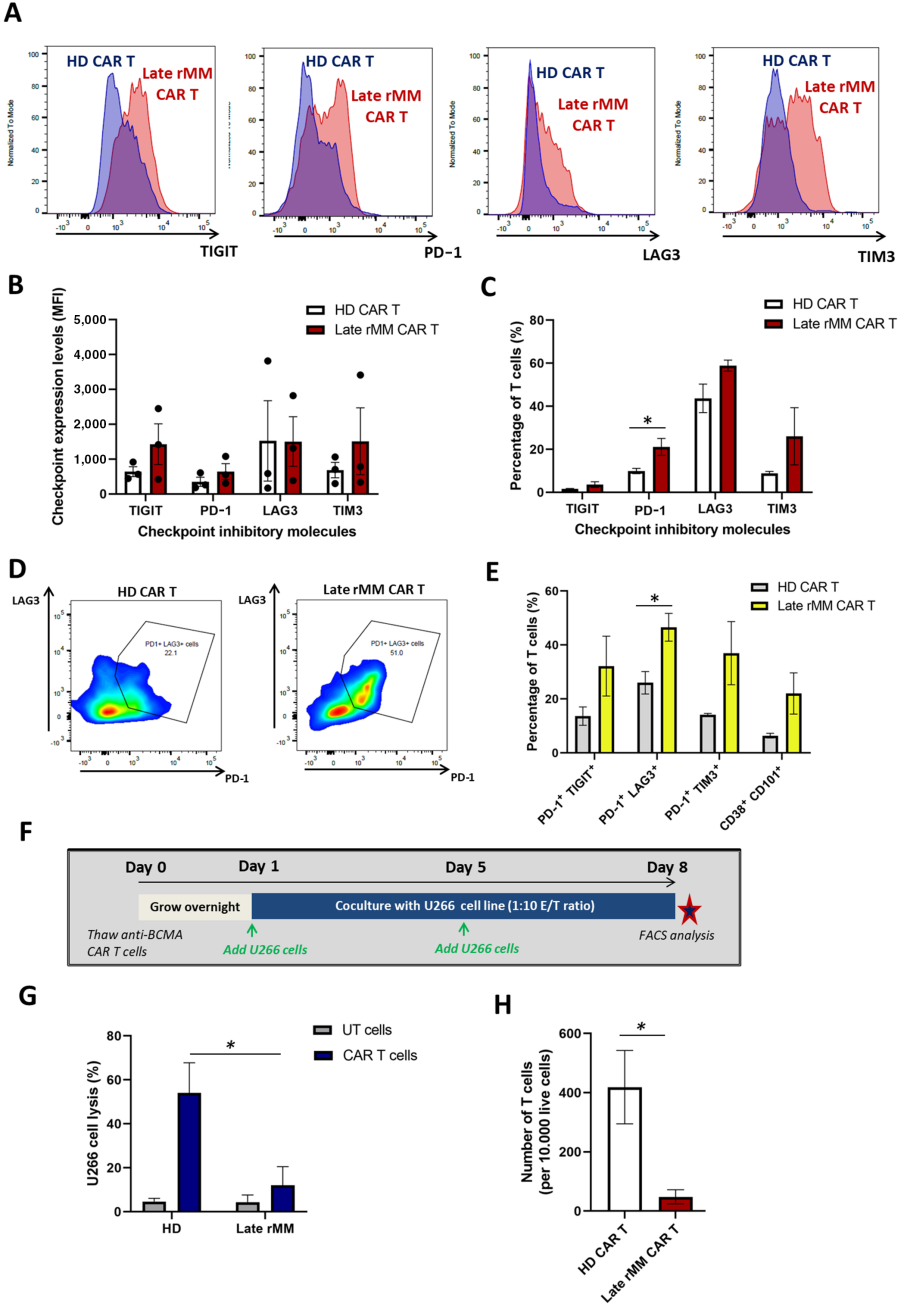
HD-derived anti-BCMA CAR T cells express lower levels of PD-1, TIGIT and LAG3 compared with multiple myeloma–derived anti-BCMA CAR T cells and show superior cytotoxicity in a rechallenge assay. **A,** Representative histograms comparing the expression of the T-cell checkpoint inhibitory molecules TIGIT, PD-1, LAG3, and TIM3 on HD-derived (blue) versus multiple myeloma (MM)-derived (red) anti-BCMA CAR T cells, analyzed by FACS at the end of production (day 14, before cryopreservation). **B,** Quantitation of MFI of TIGIT, PD-1, LAG3, and TIM3 across HD-derived (*n* = 3) and late rMM-derived (*n* = 3) anti-BCMA CAR T-cell samples. **C,** Percentage of TIGIT^+^, PD-1^+^, LAG3^+^, and TIM3^+^ on anti-BCMA CAR T cells from HD (*n* = 3) versus late rMM patients (*n* = 3) at the end of production (day 14). **D,** Representative density plots depicting the expression of the checkpoint inhibitory molecules PD-1 and LAG3 on HD-derived versus late rMM–derived anti-BCMA CAR T cells, analyzed by FACS and gated on PD-1^+^LAG3^+^ double expression. **E,** Percentage of HD-derived versus late rMM–derived anti-BCMA CAR T cells expressing PD-1^+^TIGIT^+^, PD-1^+^LAG3^+^, PD-1^+^TIM3^+^ and the permanently dysfunctional CD38^+^CD101^+^ T-cell population. **F,** Schematic diagram describing the cytotoxicity rechallenge assay consisting of a prolonged coculture of anti-BCMA CAR T cells and the multiple myeloma cell line U266, using a 1:10 E/T ratio. U266 cells were added to the anti-BCMA CAR T cells twice, on days 1 and 5. The coculture assay wells were analyzed by FACS on day 8. UT cells were used to measure T-cell background killing. **G,** Percentage of U266 cell lysis (day 8) when cocultured with HD-derived UTs (*n* = 3) or HD-derived anti-BCMA CAR T cells (*n* = 3) versus late rMM–derived UTs (*n* = 3) or late rMM–derived anti-BCMA CAR T cells (*n* = 3), analyzed by FACS using the viability dye e450. **H,** T-cell expansion of HD-derived (*n* = 3) versus late rMM–derived (*n* = 3) anti-BCMA CAR T cells at the end of the rechallenge assay (analyzed by FACS). Data represent mean values ± SEM. *P < 0.05. Statistical analysis was performed using two-tailed unpaired *t* test.

Given that significant differences were observed between HD and late relapsed multiple myeloma populations in the above analysis, and late relapsed multiple myeloma is the most relevant group for CAR T-cell treatment, we assessed the expression of the immune checkpoint molecules PD-1, TIGIT, LAG3, and TIM3 on HD-derived and late relapsed multiple myeloma-derived CAR T cells by flow cytometry. As shown in Fig. 3A–B, PD-1, TIGIT, TIM3, and LAG3 expression by MFI was not significantly different between HD and late relapsed multiple myeloma CAR T-cell products. However, the percentage of PD-1–expressing CAR T cells was significantly increased in late relapsed multiple myeloma compared with HD CAR T-cell products (Fig. 3C, *P* < 0.05). Coexpression of immune checkpoints on T cells was also compared between HD and late relapsed multiple myeloma CAR T-cell products. Trends toward higher double positivity were noted, only achieving significance for PD-1^+^LAG3^+^ cells (46.57 ± 5.1%) in late relapsed multiple myeloma compared with HD CAR T cells (26.0 ± 4.2%, *P* < 0.05; Fig. 3D and E). CD38^+^CD101^+^ T cells, which characterize a permanently dysfunctional population of T cells, previously linked to post-stem cell transplant relapse in multiple myeloma (33), were present in 22.02 ± 7.6% of late relapsed multiple myeloma CAR T cells compared with 6.39 ± 0.9% in HD products (NS; Fig. 3E).

The cytotoxic properties of HD and late relapsed multiple myeloma anti-BCMA CAR T cells were compared using a 7-day *in vitro* rechallenge assay with U266 cells as the target (Fig. 3F). HD CAR T cells showed superior cytotoxicity compared with late relapsed multiple myeloma CAR T cells (54% vs. 12% target cell lysis, *P* < 0.05) and greater expansion against U266 cells (418.5 ± 124 vs. 48.4 ± 24 T cells, *P* < 0.05; Fig. 3G and H).

### HD-Derived Anti-BCMA CAR T Cells Efficiently Target Multiple Myeloma Primary Cells in *Ex Vivo* Assays including BM Microenvironment Constituents from Patients with Multiple Myeloma

**FIGURE 4:**
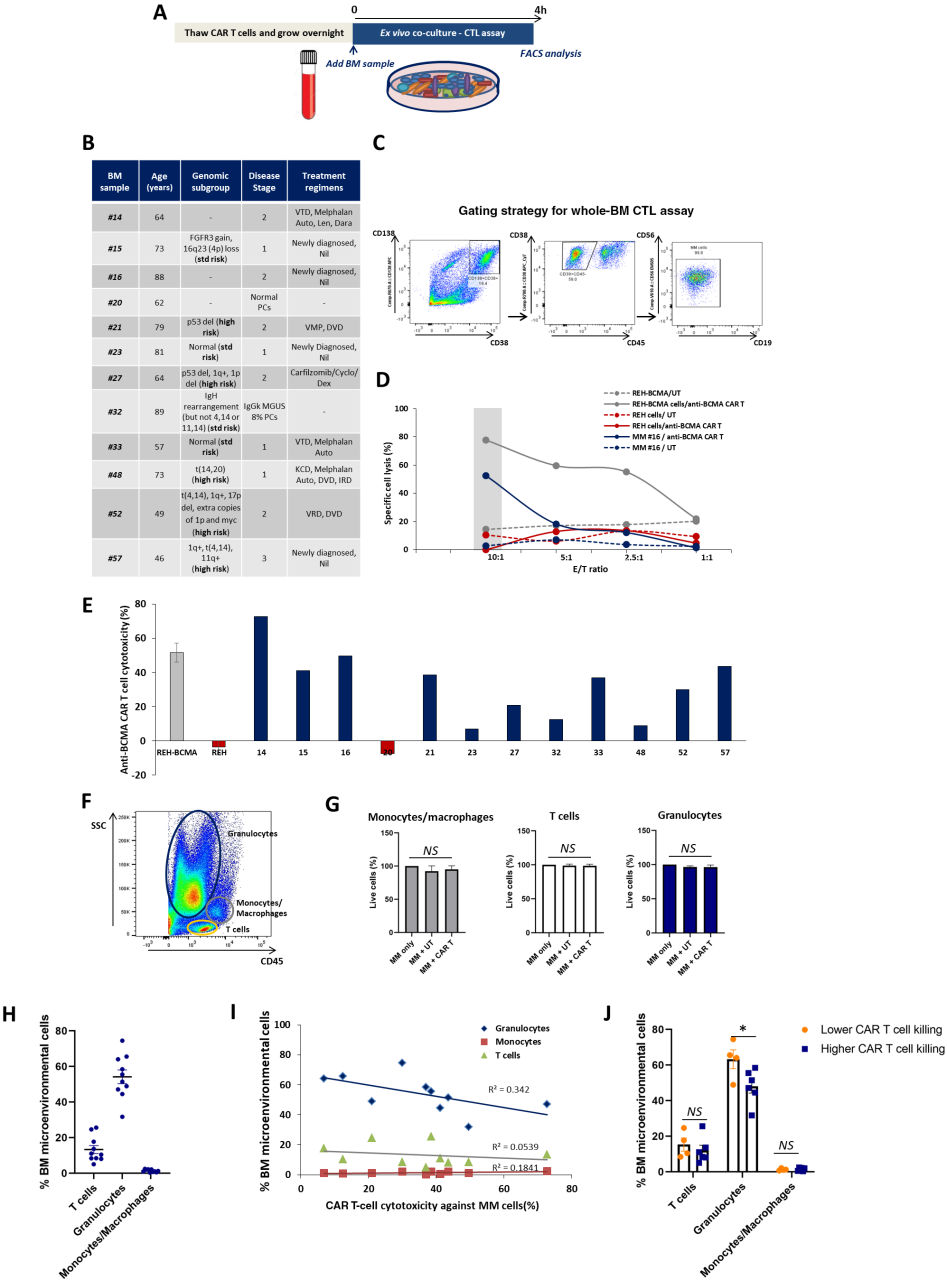
HD-derived anti-BCMA CAR T cells specifically and efficiently target multiple myeloma (MM) primary cells within the BM microenvironment of different patient subgroups. **A,** Schematic timeline of the *ex vivo* cytotoxicity (CTL) assay using BM samples from patients with multiple myeloma to test the activity of HD-derived anti-BCMA CAR T cells. The coculture was performed for 4 hours at different E/T ratios. BM samples were always used fresh and the available volume varied from sample to sample, which limited the number of samples used in specific assays. **B,** Table describing the clinical features of the patients with multiple myeloma used in this assay (*n* = 11), including patient age, genomic subgroup, disease stage, and prior treatment regimens. BM sample #20 is an age-matched normal plasma cell control with no detectable multiple myeloma cells in the BM. **C,** Representative dot plots describing the gating strategy used by FACS to identify multiple myeloma cells within the BM sample after staining with the multiple myeloma antibody panel. Multiple myeloma cells are sequentially gated as CD138^+^/CD38^hi^, CD38^hi^/CD45^−^ and CD56^+^/CD19^−^. **D,** Specific cancer cell lysis of MM#16 primary cells after coculture with anti-BCMA CAR T cells (continuous line) or UT cells (dotted line) at different E/T ratios. REH cell line and REH-BCMA^+^ cell line were used as negative and positive controls, respectively. UT cells were used to measure background T-cell killing. **E,** Specific anti-BCMA CAR T-cell killing (%) against different BM samples (*n* = 12) after the *ex vivo* CTL assay at 10:1 E/T ratio. The anti-BCMA CAR T-cell killing percentage was quantified as: [(%multiple myeloma cell lysis cocultured with anti-BCMA CAR T cells − % multiple myeloma cell lysis cocultured with UT cells) / % spontaneous multiple myeloma cell lysis]. REH and REH-BCMA were used as negative and positive controls, respectively. **F,** Representative FACS plot of a BM sample using the SSC/CD45 gating strategy to identify the different BM cell types present, including: granulocytes, monocytes/macrophages, and T cells. **G,** Cell viability of monocytes/macrophages, T cells, and granulocytes present within the multiple myeloma BM samples (*n* = 11) after the *ex vivo* CTL assay with UT cells or anti-BCMA CAR T cells. **H,** Percentage of BM microenvironmental cells (T cells, granulocytes, and macrophages/monocytes) present in the multiple myeloma BM samples prior to the *ex vivo* CTL assay (*n* = 10). **I,** Correlation between % BM microenvironmental cells and CAR T-cell cytotoxic activity for each multiple myeloma sample (*n* = 10). **J,** Percentage of BM microenvironmental cells (T cells, granulocytes, and macrophages/monocytes) present in samples with lower CAR T-cell killing (below CAR T-cell killing average of 32.9%) versus higher CAR T-cell killing (above CAR T-cell killing average of 32.9%). Data represent mean values ± SEM. NS, not statistically significant; *P < 0.05. Statistical analysis was performed using two-tailed unpaired *t* test. Abbreviations: Auto, Autologous stem cell transplant/autograft; Cyclo, Cyclophosphamide; Dara, Daratumumab; Dex, Dexamethasone; DVD, Daratumumab, Velcade, Dexamethasone; IRD, Ixazomib, Lenalidomide, Dexamethasone; Len, Lenalidomide; KCD, Carfilzomib, Cyclophosphamide, Dexamethasone; Nil, no prior treatment regimen; VMP, Velcade, Melphalan, Prednisolone; VTD, Velcade, Thalidomide, Dexamethasone.

To evaluate the activity of HD-derived anti-BCMA CAR T cells in a clinically relevant model, we designed a whole BM *ex vivo* cytotoxicity assay using BM samples from patients with multiple myeloma to reflect the immunosuppressive effects of the BM tumor microenvironment (Fig. 4A). We tested 11 multiple myeloma samples from different patient subgroups (including those with standard and high-risk cytogenetics), age, disease stage, and prior treatment regimens as well as one age-matched normal control (MM#20; Fig. 4B). Primary multiple myeloma cells were identified within the BM using a gating strategy as described in Fig. 4C and ranged from 1% to 16% of total nucleated cells.

HD-derived anti-BCMA CAR T cells efficiently targeted multiple myeloma primary cells in a dose–response manner as shown in the representative example (Fig. 4D), with 52.5% cell lysis of MM#16 at an E:T ratio of 10:1 compared with 2.8% lysis using UT cells from the same donor. Across the different multiple myeloma samples (*n* = 11), the specific tumor lysis of HD-derived anti-BMCA CAR T cells ranged from 6.9% to 72.8% (Fig. 4E). The specificity of anti-BCMA CAR T cells toward multiple myeloma primary cells was confirmed by demonstrating no significant lysis of granulocytes, macrophages/monocytes, or T cells in the BM samples following coculture (Fig. 4F and G).

The impact of the tumor microenvironment on CAR T-cell function was investigated by assessing the relationship between CAR T cell–mediated multiple myeloma cell lysis and the proportion of granulocytes, T cells, and monocytes/macrophages in BM (Fig. 4H–J). No direct correlation between T cells, monocyte/macrophage, or granulocyte percentage and tumor lysis was observed (Fig. 4I). However, there was a significant difference in granulocyte percentage (63% vs. 48%, *P* < 0.05) between BM samples shown to have low versus high tumor lysis (defined as tumor lysis below or above mean of 32.9%; Fig. 4J).

### Anti-BCMA CAR T-Cell Cytotoxicity Does Not Correlate with BCMA Expression on Primary Multiple Myeloma Cells but can be Enhanced by Treatment with GSIs

**FIGURE 5:**
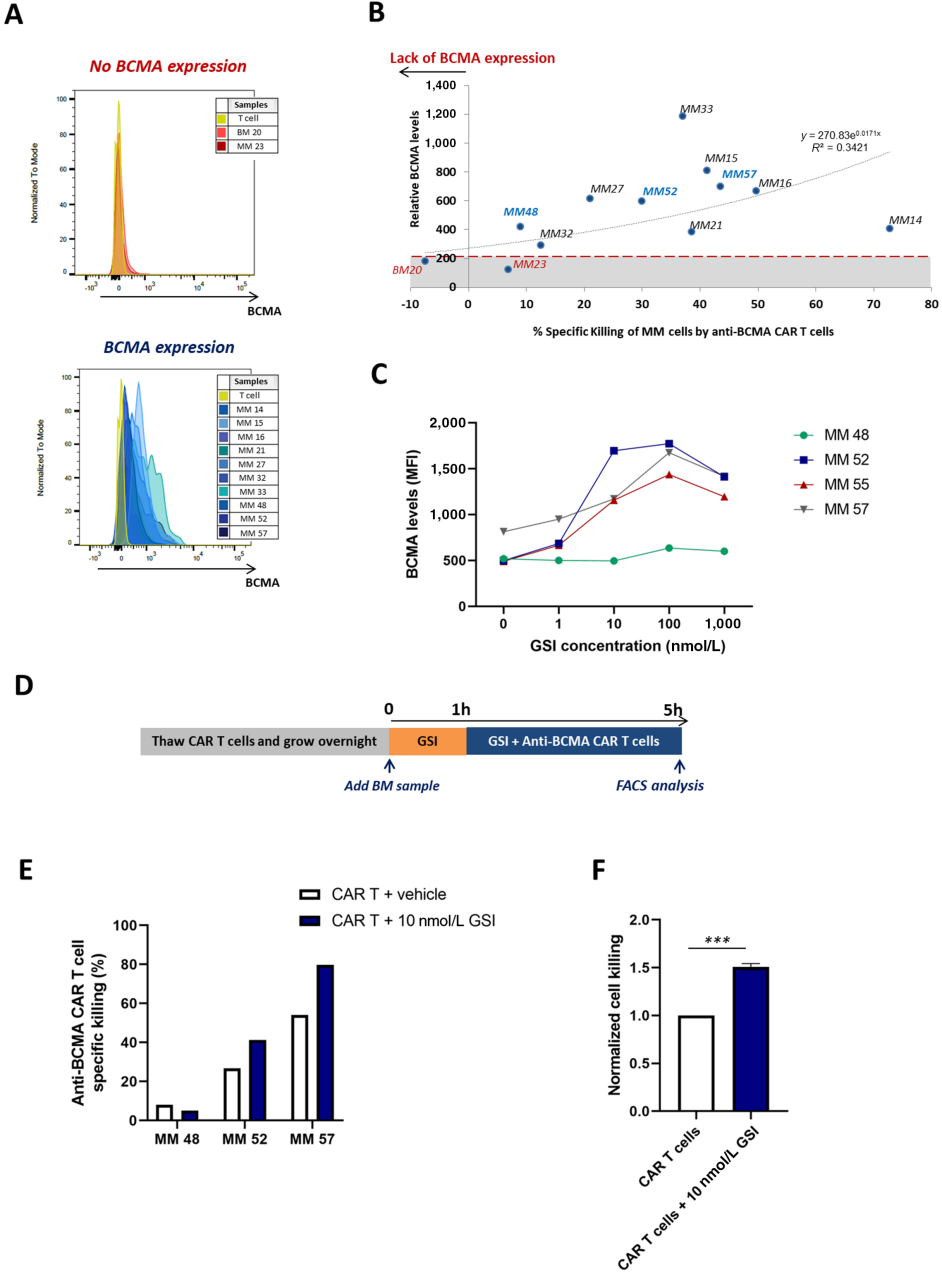
Anti-BCMA CAR T-cell activity can be enhanced by increasing BCMA levels on the multiple myeloma (MM) cell surface. **A,** Histograms showing the BCMA expression of multiple myeloma primary cells from each BM sample, analyzed by FACS. The age-matched normal BM20 control and the MM23 primary cells are BCMA^−^ according to a T-cell internal control. All the other multiple myeloma samples express BCMA at varying levels. **B,** Correlation between specific anti-BCMA CAR T-cell activity and BCMA expression for each BM sample analyzed (*n* = 12). The relative BCMA expression (mean fluorescence intensity, MFI) was normalized to the BCMA expression on BM endogenous T cells, used as an internal negative control. **C,** Expression levels of BCMA (MFI) on multiple myeloma primary cells (MM48, MM52, MM55, MM57) after treatment with GSI PF-03084014 for five hours at different concentrations. **D,** Diagram illustrating the experimental timeline used to combine the treatment of GSI PF-03084014 with anti-BCMA CAR T cells in the *ex vivo* CTL assay with multiple myeloma BM samples. BM samples were always used fresh and the available volume varied from sample to sample, which limited the number of samples used in specific assays. **E,** Specific killing of MM48, MM52, and MM57 cancer cells by anti-BCMA CAR T cells or UT cells, with and without GSI treatment. **F,** Normalized cell killing of anti-BCMA CAR T cells against MM52 and MM57 primary cells (*n* = 2) with or without 10 nmol/L of GSI treatment. Values normalized to the specific killing of anti-BCMA CAR T cells only. ***P < 0.005. Statistical analysis was performed using one-tailed paired *t* test.

Relative BCMA expression on primary myeloma cells (BCMA MFI on primary myeloma cells / BCMA MFI on T cells), ranged from 121 to 1,184 with a median of 549 (Fig. 5A). No direct correlation was found between relative BCMA expression on multiple myeloma cells and CAR T cell–mediated killing (*R*^2^ = 0.34; Fig. 5B). BCMA is actively cleaved from the surface of multiple myeloma cells by the gamma secretase complex which results in reduced target density and could potentially lead to decreased recognition and anti-BCMA CAR T-cell activity. Treatment of multiple myeloma primary samples with the GSI PF-03084014 at concentrations of 1 to 1,000 nmol/L significantly increased BCMA expression at the multiple myeloma cell surface in 3 of 4 samples with a median rise in MFI from 601 to 1,381 after treatment with GSI at 100 nmol/L (Fig. 5C). To evaluate the effect of GSI treatment on anti-BCMA CAR T-cell activity, we treated BM samples with GSI and anti-BCMA CAR T cells (Fig. 5D). The combination of 10 nmol/L of GSI with allogeneic anti-BCMA CAR T cells led to a 1.54-fold increase in killing of MM#52 primary cells (41.3% vs. 26.8%) and 1.48-fold increase for MM#55 primary cells (79.7% vs. 54.1%) when compared with vehicle-treated controls (Fig. 5E), but not in MM#48 (5.15% vs. 8.14%). In BM samples where BCMA expression increased after GSI treatment, a synergistic effect was seen between GSI and anti-BCMA CAR T cells compared with anti-BCMA CAR T cells alone (*P* < 0.005, Fig. 5F).

## Discussion

Treatment with autologous anti-BCMA CAR T cells results in high overall response rates and deep remissions in relapsed refractory MM with a median duration of response between 8.8 and 22 months (19–21, 34, 35). Impaired T-cell fitness in patient-derived CAR T cells and an immunosuppressive BM microenvironment are thought to be the main causes for multiple myeloma relapse post CAR T-cell treatment. Patients with multiple myeloma are generally older with a median age of 72 years and have usually had multiple lines of treatment prior to being considered for CAR T-cell therapy. Both these factors have the potential to affect the fitness of their T cells. An alternative source of CAR T cells are young HDs (allogeneic) whose T-cell fitness is unlikely to be impaired. Allogeneic CAR T cells offer the opportunity for patients with low T-cell counts who fail to generate an autologous CAR T-cell product to still be treated. In this study, we therefore compared the phenotype and antitumor activity of anti-BCMA CAR T cells derived from patients with relapsed refractory multiple myeloma with those derived from young HDs.

HDs were shown to have higher T-cell counts compared with patients with relapsed multiple myeloma. Furthermore, HDs were found to have a significantly higher CD4/CD8 T-cell ratio than patients with late relapse multiple myeloma. In contrast, patients with untreated newly diagnosed multiple myeloma had a similar CD4/CD8 ratio to HDs suggesting that prior antimyeloma treatment may have an impact on the CD4/CD8 ratio. A decrease in CD4/CD8 ratio has previously been described in patients with multiple myeloma after treatment with daratumumab (36) and bortezomib (37). Dexamethasone has also been associated with lower CD4/CD8 ratio in premature infants (38). A higher CD4/CD8 T-cell ratio in the leukapheresis product has previously been shown to correlate with greater expansion of anti-BCMA CAR T cells *in vivo* (19, 24) and better clinical responses (24). In line with these findings, some groups have modified their manufacturing process to generate CAR T-cell products with a predefined CD4/CD8 T-cell ratio (39) (NCT03430011) but it remains unclear whether this will result in improved clinical outcomes.

T-cell memory is also directly affected by age and MM treatment (36, 40) and high levels of naïve and stem cell memory T cells in the leukapheresis product have been associated with higher expansion of anti-BCMA CAR T cells *in vivo* and better clinical responses (24, 26). Our data demonstrates that at baseline HDs have a higher percentage of naïve T cells and lower proportion of effector T cells compared with patients with newly diagnosed multiple myeloma and relapsed multiple myeloma and importantly, postmanufacturing, have a higher percentage of central memory T cells in the final CAR T-cell product.

Clinical trials also suggest that high levels of exhaustion markers in autologous anti-BCMA CAR T cells correlate with poor clinical responses in patients with multiple myeloma (26). A detailed analysis of CAR T cells from patients with non-responder multiple myeloma revealed that these autologous products were mainly formed by activated effector T cells that expressed the checkpoint inhibitory markers, LAG3 and TIM3 (41). In fact, the expression of the checkpoint inhibitory molecules PD-1, TIM3, LAG3, and TIGIT has long been associated with lack of T-cell fitness and increased incidence of multiple myeloma relapse (33, 42–45). We showed that PD-1^+^ T cells were significantly enriched in late relapsed multiple myeloma-derived CAR T cells compared with HD-derived products, which may promote T-cell exhaustion and compromise their cytotoxic ability. PD-1^+^LAG3^+^ double positive cells were also present in significantly higher numbers in late relapsed multiple myeloma-derived CAR T cells compared with HD-derived CAR T cells. These dysfunctional late relapsed multiple myeloma–derived CAR T cells were shown to proliferate less well and have reduced cytotoxicity against U266 multiple myeloma cells *in vitro* in comparison with HD-derived CAR T cells, which had a less exhausted phenotype.

For allogeneic CAR T cells to be used in the clinic there would need to be further genetic modification, for instance knockout of the endogenous T-cell receptor (*TCR*) and *CD52* genes to prevent graft-versus-host disease and allow the use of an anti-CD52 antibody as part of lymphodepletion to prevent CAR T-cell rejection, respectively (28). Of note HD-derived CAR T cells used in this study had not undergone such genetic manipulation; however, we do not anticipate these gene editing modifications to significantly alter the T-cell fitness of the final CAR T-cell product (28). In the ongoing UNIVERSAL trial of *TCR*^KO^/*CD52*^KO^ HD-derived anti-BCMA CAR T cells (ALLO-715) in patients with multiple myeloma, there was evidence of CAR expansion in blood and encouraging clinical responses (NCT04093596) (46). *TCR*^KO^ HD-derived anti-CD19 CAR T cells were previously shown to have superior functionality over lymphoma patient-derived CAR T cells (47). Furthermore clinical trials of allogeneic HD-derived *TCR*^KO^/*CD52*^KO^ anti-CD19 CAR T cells (UCART19) in relapsed refractory B-acute lymphoblastic leukemia demonstrated significant CAR expansion and impressive clinical responses (48, 49) supporting the hypothesis that CAR T cells derived from HDs may have superior functionality to products made from patients. Persistence of allogeneic CAR T cells, however, appears to be of shorter duration than with autologous CAR T cells presumably due to host immune system–mediated rejection (49). Graft-versus-host disease was rarely seen in these early trials.

Another important factor that may contribute to multiple myeloma relapse is the presence of multiple myeloma niches within the immunosuppressive environment of the BM. Multiple myeloma cells are known to be highly dependent on the BM microenvironment and the physical interaction between multiple myeloma cells and BM cells is known to promote cancer resistance against different therapeutics (50, 51). Having demonstrated cytotoxicity against BCMA-positive myeloma cell lines *in vitro*, we evaluated the efficacy of HD-derived anti-BCMA CAR T cells in a clinically relevant model using *ex vivo* whole BM biopsies from patients with multiple myeloma, to take into account the genomic complexity and immunosuppressive cellular environment seen in these patients. We showed that HD-derived anti-BCMA CAR T cells specifically target multiple myeloma primary cells *ex vivo* demonstrating activity against primary multiple myeloma cells with a range of different genomic abnormalities. No differences were seen among the genomic risk subgroups, which corroborates what has been described across anti-BCMA CAR T-cell clinical trials (19, 20, 24, 26). With the exception of tumor burden, anti-BCMA CAR T-cell activity did not correlate with any other biological factor intrinsic to multiple myeloma primary cells, such as expression of CD38, CD138, CD56, or CD45. Importantly, we showed no correlation between the level of BCMA expression on multiple myeloma primary cells and anti-BCMA CAR T cell–mediated cytotoxicity. This is in keeping with other studies that have shown no correlation between baseline BCMA expression on multiple myeloma cells and clinical response to anti-BCMA CAR T-cell therapy (19, 20, 24).

Taking advantage of our *ex vivo* BM model, we explored whether the presence of other BM microenvironmental cells affects anti-BCMA CAR T-cell activity. An interesting observation in our study was the finding of significantly higher granulocyte numbers in BM samples where low CAR T-cell cytotoxicity was seen compared with BM samples with high cytotoxicity, although a direct correlation between granulocyte numbers and cytotoxicity was not observed. Neutrophils, a subset of granulocytes, have been associated with cancer progression, metastasis, and poor prognosis in solid tumors (52). Tumor-associated neutrophils are known to express checkpoint inhibitory molecules such as PD-L1 and CD86 that can directly inhibit T cells (53, 54), and a dysfunctional neutrophil profile has been detected in patients with multiple myeloma (55–57). In addition, high levels of neutrophils at diagnosis and high neutrophil to lymphocyte ratio are associated with poor prognosis and overall survival in patients with multiple myeloma (56–58). Further evaluation is therefore needed to clarify whether granulocytes or a particular subset of granulocytes may have a negative impact on anti-BCMA CAR T-cell function.

BCMA loss has also been shown to be a mechanism of relapse post anti-BCMA CAR T-cell therapy due to clonal selection of low antigen–expressing cells, allelic deletion or mutations of the *BCMA* gene (24–26, 59). BCMA expression is a highly dynamic process and is regulated by the protease gamma secretase, which releases soluble BCMA into the bloodstream (60). We investigated, in our *ex vivo* BM assay, combination treatment of multiple myeloma with allogeneic anti-BCMA CAR T cells and a GSI in an attempt to transiently increase BCMA expression at the cell surface of multiple myeloma cells and make them more amenable to targeting by CAR T cells. With this strategy we were able to show upregulation of BCMA expression on primary multiple myeloma cells in a majority of samples and, importantly, higher cytotoxicity. This combination strategy has been used by others in the context of autologous anti-BCMA CAR T cells (ref. 61; NCT03502577) and could also be explored with allogeneic anti-BCMA CAR T cells.

In summary, we have shown that HD-derived anti-BCMA CAR T cells have a distinct immune phenotype and superior long-term *in vitro* activity compared with relapsed multiple myeloma–derived CAR T cells. Our data lend support to the use of allogeneic HD CAR T cells as an alternative therapeutic option especially for patients with relapsed multiple myeloma with poor T-cell counts following multiple lines of treatment and in the setting of autologous CAR T-cell manufacturing failure. Clinical trials with allogeneic anti-BCMA CAR T cells in relapsed multiple myeloma are ongoing.
